# The Cost of Toxicity in Microalgae: Direct Evidence From the Dinoflagellate *Alexandrium*

**DOI:** 10.3389/fmicb.2019.01065

**Published:** 2019-05-22

**Authors:** Hannah E. Blossom, Bo Markussen, Niels Daugbjerg, Bernd Krock, Andreas Norlin, Per Juel Hansen

**Affiliations:** ^1^ Marine Biological Section, Department of Biology, University of Copenhagen, Elsinore, Denmark; ^2^ Department of Mathematical Sciences, University of Copenhagen, Copenhagen, Denmark; ^3^ Marine Biological Section, Department of Biology, University of Copenhagen, Copenhagen, Denmark; ^4^ Alfred-Wegener Institut für Polar-und Meeresforschung, Bremerhaven, Germany

**Keywords:** toxicity, *Alexandrium*, costs, PST, allelochemicals, lytic compounds, harmful algae

## Abstract

Empirical evidence of the cost of producing toxic compounds in harmful microalgae is completely lacking. Yet costs are often assumed to be high, implying substantial ecological benefits with adaptive significance exist. To study potential fitness costs of toxin production, 16 strains including three species of the former *Alexandrium tamarense* species complex were grown under both carbon limitation and unlimited conditions. Growth rates, levels of intracellular paralytic shellfish toxins (PSTs), and effects of lytic compounds were measured to provide trade-off curves of toxicity for both PST and lytic toxicity under high light (300 μmol photons m^−2^ s^−1^) and under low light (i.e., carbon limited; 20 μmol photons m^−2^ s^−1^). Fitness costs in terms of reduced growth rates with increasing PST content were only evident under unlimited conditions, but not under carbon limitation, in which case PST production was positively correlated with growth. The cost of production of lytic compounds was detected both under carbon limitation and unlimited conditions, but only in strains producing PST. The results may direct future research in understanding the evolutionary role and ecological function of algal toxins. The intrinsic growth rate costs should be accounted for in relation to quantifying benefits such as grazer avoidance or toxin-mediated prey capture in natural food web settings.

## Introduction

The microalgae responsible for harmful algal blooms (HABs) produce a wide variety of bioactive natural products, or secondary metabolites, known as phycotoxins (Smayda, 1997; Cembella, 2003). The best studied of these toxins are those that accumulate in the tissue of shellfish and fish and cause intoxications further in the food chain through consumption by predators; these toxins may at times impose a human health threat, i.e., paralytic shellfish toxins (PSTs), diarrhetic shellfish toxins (DSTs), amnesic shellfish toxins (ASTs), and ciguatoxins (Anderson et al., 2012). Despite the structural identification of hundreds of these toxins and their analogs, a comprehensive understanding of the functional and adaptive significance of toxin production is unknown. Many have suggested that these compounds serve a defensive role (Smetacek, 2001; Selander et al., 2006), yet overall, the collective results of previous research (Turner, 2006, 2014) do not provide unequivocal evidence that the primary function of shellfish toxins is predation defense (Cembella, 2003; Anderson et al., 2012). In fact, it is possible that each compound serves a different function, as these are chemically diverse compounds, found in a variety of taxonomic groups.

Many HAB species also produce toxins, referred to as lytic compounds or allelochemicals, which can be released into the surrounding environment, where they cause cell lysis and death of other planktonic organisms. The chemical structures of these toxins are still, in most cases, unresolved, exceptions being polyethers like, karlotoxins, karmitoxins, and prymnesins (Van Wagoner et al., 2008, 2010; Rasmussen et al., 2016, 2017). In contrast to the known phycotoxins previously mentioned, the lytic compounds have a more direct ecological role as toxins because they cause cell lysis of algal competitors and protist grazers. This chemically induced interference competition is a concept known as allelopathy, well described in terrestrial plants (Molish, 1937). Yet in an aqueous medium governed by dilution, turbulence, and viscous forces, a released compound may never reach the intended target in relevant concentrations, and thus, a threshold density of lytic algae must be reached to elicit an effect (Jonsson et al., 2009). Because of this and the potential for non-toxic individuals to receive a benefit, without suffering the cost of producing lytic compounds, the selective driving forces for allelopathy as an adaptive competitive strategy have been challenged (Lewis, 1986; Jonsson et al., 2009). Like the known phycotoxins, the evolutionary role and ecological function of lytic compounds have become unclear.

The continual production and release of unknown lytic toxins, which may be complex metabolites requiring intricate biosynthesis pathways to produce, while avoiding self-toxicity, is often assumed, and widely stated in the literature, to be costly (Wolfe, 2000; Legrand et al., 2003; Gross et al., 2012). If toxicity can be considered an adaptive trait, in the absence of sufficient benefits, a high cost of toxin production would increase the relative fitness of the non-toxic individuals in comparison. Thus, conspicuous ecological effects, like competitive exclusion and grazer defense, have been interpreted as significant, adaptive benefits. This high cost of toxicity, however, could be challenged (John and Flynn, 2002; Pohnert, 2009; Ianora et al., 2011). If toxins are produced as a result of another trait or process, like the passive process of maintaining optimal intracellular nutrient ratios, then the costs might be small and insignificant compared to measureable benefits (Ianora et al., 2006; Glibert et al., 2016; Berge et al., 2017). Although the few studies that have addressed potential costs only used one or two strains with unlimited conditions, no evidence of costs were detected (Bergkvist et al., 2008; Tillmann et al., 2009; John et al., 2015). Thus, there is a need to measure the costs and benefits of toxicity experimentally.

The study presented here focuses on one of the most notorious HAB dinoflagellates, members of the genus *Alexandrium.* Species of *Alexandrium* are responsible for the most severe and lethal of the shellfish poisonings, paralytic shellfish poisoning, due to the production of the potent alkaloid neurotoxin saxitoxin and its derivatives, collectively known as paralytic shellfish toxins (PSTs). *Alexandrium* species are also capable of releasing potent compounds, which have lytic effects on a variety of other protists and grazers (Hansen et al., 1992; Tillmann and John, 2002; Tillmann and Hansen, 2009), impacting the plankton community structure (Fistarol et al., 2004). The lytic compounds of *Alexandrium* are distinct from the PSTs, as some lytic *Alexandrium* species do not produce PSTs (Tillmann and John, 2002), and the strength of the lytic effect is not correlated to levels of PSTs among different strains (Arzul et al., 1999; Tillmann and John, 2002; Fistarol et al., 2004; Hattenrath-Lehmann and Gobler, 2011).

Here, we took a trait-based approach and quantified trade-offs of both PST and lytic compound production in members of the former *Alexandrium tamarense* species complex, by measuring growth rates, PST content, and lytic toxicity in 16 strains. Costs may arise, through resource allocation, when the resources of a cell are allocated toward toxin production and are therefore unavailable for primary metabolic processes like reproduction and growth. Under limiting conditions, the microalgae will be faced with a trade-off: either invest more in growth, and less in toxicity, or maintain investments in toxicity, but suffer a greater reduction in growth rate. Here, the strains (in triplicate) were subject to both carbon limitation (caused by low light) and unlimited conditions (high light); therefore, photosynthetic carbon uptake and cellular carbon content were also measured. Assuming variability in toxicity among strains under both light conditions, we hypothesize that if the cost of toxin production (and excretion, in the case of lytic compounds) is high, there will be a negative relationship between toxicity and growth, specifically under limiting conditions. If the cost of production is low or negligible, there will be no relationship between toxicity and growth.

## Materials and Methods

### Algal Strains, Species Identification, and Culture Conditions

The 16 *Alexandrium* strains used in this study were chosen because of their potential to produce toxins as they all belong to the former *A. tamarense* species complex (Scholin et al., 1994). The strains came from various culture collections including the Culture Collection of Algae and Protozoa (CCAP-strains), the Norwegian Culture Collection of Algae (K-1490), the National Center for Marine Algae and Microbiota (NCMA; CCMP-strains), and the Roscoff Culture Collection (RCC strains). Urban Tillmann kindly supplied the strains Alex2, Alex5, and H5. Detailed information about the strains can be found in Table 1.

**Table 1:** Strains of *Alexandrium* species examined in this study with identification based on LSU rDNA ribo-types, the original assigned name their strain name in culture collections or private collections, the geographical location, and the year of isolation.

Molecular-based species identification	Species name in culture collection	Strain name	Geographic location	Isolation year	Genbank accession number
*A. catenella*	*A. fundyense*	CCMP-1719	New Hampshire, USA	1985	MK566196
*A. catenella*	*A. fundyense*	CCMP-1979	Bay of Fundy, USA/Canada	1998	MK566197
*A. catenella*	*A. fundyense*	RCC-4086	Massachusetts, USA	2001	MK566193
*A. catenella*	*A. tamarense*	CCAP-1119/32	Scotland	2008	MK566200
*A. catenella*	*A. tamarense*	CCAP-1119/27	Scotland	2008	MK566194
*A. catenella*	*A. catenella*	Alex2	Scotland	2009	MK566201
*A. catenella*	*A. catenella*	Alex5	Scotland	2009	MK566198
*A. catenella*	*A. catenella*	SCCAP K-1490	Canada	2009	MK566195
*A. catenella*	*A. catenella*	H5	Argentina	2012	MK566199
*A. pacificum*	*A. catenella*	CCAP-1119/52	France	Unknown	MK566191
*A. pacificum*	*A. tamarense*	CCMP-1493	Hong Kong Island, China	1991	MK566192
*A. pacificum*	*A. tamarense*	CCMP-1598	Hong Kong Island, China	1991	MK566190
*A. tamarense*	*A. tamarense*	CCMP-1771	England	1957	MK566187
*A. tamarense*	*A. tamarense*	RCC-4087	Sweden	1991	MK566186
*A. tamarense*	*A. tamarense*	CCAP-1119/33	Scotland	2007	MK566188
*A. tamarense*	*A. tamarense*	CCAP-1119/20	Scotland	2008	MK566189

In the remaining text, CCAP strains will be referred to without “1119/,” but the complete strain name given by the culture collection is shown here.

A trait-based approach was used here, and we selected strains that would likely provide a data set with a broad range of both lytic toxicity and PST content, including strains lacking toxicity; thus, the species designations of the strains used were not the focus of this study. However the taxonomy of *Alexandrium* species belonging to the former *A. tamarense* species complex has recently been revised (John et al., 2014a), which resulted in an emendation of five species previously assigned to groups I-V (Lilly et al., 2007). In order to avoid misidentification, this molecular based approach (John et al., 2014a) was used here to correctly identify the 16 *Alexandrium* strains (detailed methods found in the Supplementary Materials).

Prior to experimental preparations, all cultures had been maintained at 15°C with a 14:10 light:dark cycle at 100 μmol photon m^−2^ s^−1^ in f/2 medium made with pasteurized seawater and a salinity of 30. Experimental conditions were the same except for altering the irradiance levels.

### Experimental Setup

Each strain was grown in triplicates at two irradiance treatments, 20 and 300 μmol photons m^−2^ s^−1^, referred to in the text as “low light” (LL) and “high light” (HL) treatments, respectively, for 96 total experimental units. The strains were gradually acclimated to the specific light treatment in four increasing or decreasing light increments at which they were held for at least 3–4 days each. They were then kept at the light treatment for at least 1 week prior to the experiments.

Each acclimated culture with known cell concentration was diluted to a volume of >1.5 l with fresh f/2 medium to a final concentration of approximately 150 cells ml^−1^, the exact initial cell concentration was determined by cell enumeration using Sedgewick Rafter counting chambers. To create triplicates, this initial culture was divided into three 500-ml blue-capped glass Pyrex® bottles in volumes of approximately 500 ml. The bottles were then placed randomly at either high or low light.

### Algal Growth Rates

Subsamples of each strain grown at HL and LL were taken for cell counts and fixed with 2% Lugol’s iodine every 2–3 days and once a week, respectively. At least 200 cells were counted in a Sedgewick Rafter chamber using an inverted light microscope. Growth rates were calculated using the linear parts of the semi-log plot. At each sampling, pH was measured to ensure that the other parameters were done during times when pH would not have an influence on growth rate or toxic effects.

During the exponential growth phase, other parameters were measured: cellular carbon content, photosynthesis, lytic toxicity of the supernatant, and cellular PST content. Ideally, these measurements were all performed on each strain the same day but always within 2–3 days. These measurements were taken 2–3 times during exponential growth, but only one measurement day (in triplicates) was selected for data analysis. The multiple measurements were used as an internal control, and to ensure, measurements used for analysis were taken during the exponential growth phase.

### Cellular Carbon Content

The cellular carbon content of the *Alexandrium* strains was measured in order to have accurate biomass estimates and to understand total carbon production. Cellular carbon content was measured using a Shimadzu Total Organic Carbon (TOC) analyzer. For sampling, a 55 ml subsample was taken from each triplicate culture. This was then divided into two, a filtered sample and an unfiltered sample. The unfiltered (whole-cell) algal culture was added to a glass autosampler vial containing a small magnet to ensure the culture was well-mixed during the measurement. The filtered sample was obtained by filtering the rest of the well-mixed whole cell algal culture through GF/F filters, and retaining the cell-free filtrate. Autosampler vials were filled to capacity and sealed with foil and caps. Filters, sample vials, foil, and caps were all pre-combusted at 450°C for 4 h. TOC of the filtered and unfiltered samples was measured using the non-purgeable organic carbon (NPOC) method in the TOC analyzer. Then to determine the carbon content of the cells, the TOC of the filtered sample [which is thus dissolved organic carbon (DOC)] was subtracted from the TOC of the unfiltered samples [which would be the particulate organic carbon (POC)]. Both bacteria and algal cells would contribute to the POC because of the use of GF/F filters. Therefore, the estimated carbon biomass contributed by bacteria was subtracted from the measured carbon biomass to obtain more accurate algal cellular carbon contents. Bacterial biomass was estimated by measuring bacterial abundances using flow cytometry (see Supplementary Materials) and converting to cellular carbon content using an estimated factor of 20 fg C cell^−1^ (Lee and Fuhrman, 1987). Inorganic carbon (IC) was measured for each sample at the same time as TOC measurements, which was used for calculating photosynthesis (see below). Subsamples for determining cell concentration were taken at the same time, fixed, and counted as described above.

### Photosynthetic Carbon Uptake

Photosynthetic carbon uptake measurements were done on the same day as the biomass measurements using a ^14^C isotope method. This was done to ensure that all strains were light (carbon) limited. Two 2 ml subsamples of each triplicate were taken and placed in a 20 ml glass scintillation vial. To each of these, 20 μl of NaH^14^CO_3_ stock solution was added (specific activity = 100 μCi ml^−1^, carbon 14, Centralen, Denmark). One set of vials was incubated in culture conditions, at the experimental irradiance for 3 h. The other set was wrapped in aluminum foil and incubated at culture conditions, but in complete darkness for 3 h. After incubation, 100 μl of the sample was transferred to a new vial with 200 μl phenylethylamine to check the specific activity of the medium. The remaining 1.9 ml was spiked with 2 ml of 10% glacial acetic acid in methanol to remove inorganic carbon. These vials were incubated overnight in a 60°C heat block to dry. The following day, 1.5 ml of de-ionized water was added to the vials to re-dissolve the residue. To this, 10 ml of Packard Insta-Gel Plus scintillation cocktail was added, and new caps were placed on the vials. The amount of fixed ^14^C in disintegrations per minute was measured using a Packard 1500 Tri-Carb liquid scintillation analyzer with automatic quench correction. The photosynthetic activity per cell (PA, pg C cell^−1^ h^−1^) was calculated as follows:

$$PA\;=\;\frac{DPM\;\times \;IC}{{\;}^{14}C_{\mathrm{added}}\;\times \;h\;\times \;\mathrm{cells}\;}$$

where DPM is disintegrations min^−1^, IC is the concentration of inorganic carbon (pg C ml^−1^), ^14^C_added_ is the specific activity (in disintegrations min^−1^), h is the incubation time in hours, and cells is the total number of cells in the vial, based on cell counts. IC concentrations were determined on the same cultures using the TOC analyzer.

### Toxicity

#### Lytic Toxicity

The lytic compounds of *Alexandrium* are uncharacterized and could not be measured directly. Therefore, lytic toxicity levels were quantified using a microalgal bioassay with the cryptophyte *Teleaulax acuta* as target alga (Blossom et al., 2014) to calculate the *Alexandrium* concentration causing 50% mortality of *T. acuta* (LC_50_). For each light treatment, the supernatant of each triplicate culture with known cell concentration was diluted separately (to maintain biological triplicates) to 2 ml in 20 ml glass vials in nine different concentrations to create three dose-response curves. A control was made with 2 ml of f/2 medium, in triplicate. One milliliter of *T. acuta* was added to each vial and then incubated in low light at 15°C for 3 h. After 3 h, the relative fluorescence was measured using a Trilogy Fluorometer (Turner Designs, Inc., San Jose, CA, USA), which was converted to percent mortality by comparing the relative fluorescent unit to that of the control (100% intact *T. acuta* cells). The LC_50_ of each triplicate was calculated using the *drc* package in the open source software R. For some strains, a concentration lysing 100% of the target was not obtained before the culture reached stationary phase, or the pH became too high, but an LC_50_ could still be calculated. The strains that could not reach densities lysing >0% of the target algae were considered non-lytic.

##### Lytic Toxicity Stability at Culture Conditions

A small experiment using two of the most lytic strains (H5 and Alex2) was done to test if the rate of degradation of lytic compounds was similar under both HL and LL. This was done in order to ensure accurate lytic toxicity estimates when comparing relative toxicities between HL and LL, because some toxins are more labile with light. See Supplementary Materials for detailed methods and results.

#### Paralytic Shellfish Toxicity

Subsamples were taken for PST analysis once during the exponential growth phase. A volume of 45 ml of culture, or a volume corresponding to a total of approximately 100,000 cells, was centrifuged at 3,000 × *g* for 15 min. The pellet was re-suspended in 1.5 ml of f/2 medium, transferred to a 1.5 ml Eppendorf tube, and centrifuged again at 3,000 × *g* for 4 min. Liquid was removed, and the cell pellets were stored at −20°C until analysis. PSTs were analyzed following the methods in Tillmann et al. (2016). In short, cell pellets were extracted with 0.03 M acetic acid and filtrated over 0.45 μm centrifugation filters. PSTs were determined by ion pair chromatography on a C18 stationary phase, followed by continuous post-column derivatization (periodate oxidation and cyclisation by nitric acid) and fluorescence detection (HPLC-FLD). Quantification was performed by calibration against external standard curves of the individual PSTs or enantiomeric pairs.

### Statistical Analyses

Statistical analysis was done with the open source software R (version 3.5.1). One-way ANOVAs with random effect of strain were performed to compare measured parameters (growth, toxicity, cellular carbon content, and photosynthesis), between the two light levels, in order to get an overall comparison of how strains differed between high and low light.

To quantify intrinsic trade-offs of the potential covariates PST (expressed as either Content, Production, or Fraction of total assimilated carbon), lytic toxicity (Inverse LC_50_), and cellular carbon content (Biomass) on growth, ANCOVA modeling was performed. As the measured variables have different scales and have right tailed distributions on the non-negative axis, the modeling was done on the logarithmic scale. To incorporate the effect of high and low light, producing PST or not, and lytic or not, the intercept and slope parameters in the ANCOVAs were allowed to depend on the three-way interaction between the light level, presence or absence of PST, and lytic or non-lytic. Furthermore, the random effects of strain and of triplicate (which corresponds to light nested within strain) were both included to incorporate the dependence structure. Mathematically, the statistical model is expressed as:

$$\begin{array}{l} \log\left(\mathrm{Growth}\right)\;=\;\alpha \left(\mathrm{Light,}\;PST\;\mathrm{presence,}\;\mathrm{Lytic}\right)\;\\ \;+\;\beta \left(\mathrm{Light,}\;PST\;\mathrm{presence}\right)\;\times \;\log\left(\mathrm{Inverse}\;LC_{50}\right)\;\\ \;+\;\gamma \left(\mathrm{Light,}\;\mathrm{Lytic}\right)\;\times \;\log\left(PST\right)\;\\ \;+\;\delta \left(\mathrm{Light,}\;PST\;\mathrm{presence,}\;\mathrm{Lytic}\right)\;\times \;\log\left(\mathrm{Biomass}\right)\;\\ \;+\;A\left(\mathrm{Strain}\right)\;+\;B\left(\mathrm{Light,}\;\mathrm{Strain}\right)\;+\;\mathrm{error} \end{array}$$

To make the regression line β (Light, PST presence) × log(Inverse *LC*_50_ ) disappear for non-lytic strains, the Inverse LC_50_ variable was artificially set to one for these strains. Similarly, PST was artificially set to one for the non-PST producing strains. Due to this encoding, and since there is a visually obvious difference between growth in HL and LL, the intercept term α (Light, PST presence, Lytic) was fixed in the model. The objective was to find the most parsimonious models. To achieve this, inclusion of the regression lines and their possible dependence on light, PST producing strains, and lytic strains were decided on best subset selection based on the Bayesian information criterion (BIC).

Prior to the analysis described above, principal component analysis was done to test for collinearity between the three covariates. Furthermore, the initial and the selected models were validated by plots of residuals against fitted values and normal quantile plots of residuals and of predicted random effects. Hypothesis tests were performed by likelihood ratio in the selected models, and estimated marginal means in the eight groups defined by the three categorical variables were grouped in a post hoc analysis corrected for multiple testing.

## Results

### Species Identification

The species designations of the 16 strains of *Alexandrium* were done using approximately 900 base pairs of the LSU rDNA gene including domains D1-D2. An alignment of all the 16 strains using JalView ver. 14 (Waterhouse et al., 2009) revealed that they fell into three distinct groups. By adding randomly selected but annotated sequences from John et al. (2014a), their Supplementary File S9, we were able to identify nine strains of *A. catenella*, four strains of *A. tamarense*, and three strains of *A. pacificum*. A total of eight strains had the wrong species designations and thus needed a name change, see Table 1. We followed the proposal by Fraga et al. (2015) for preferring *A. catenella* over *A. fundyense* (John et al., 2014b).

### Growth

**Figure 1:**
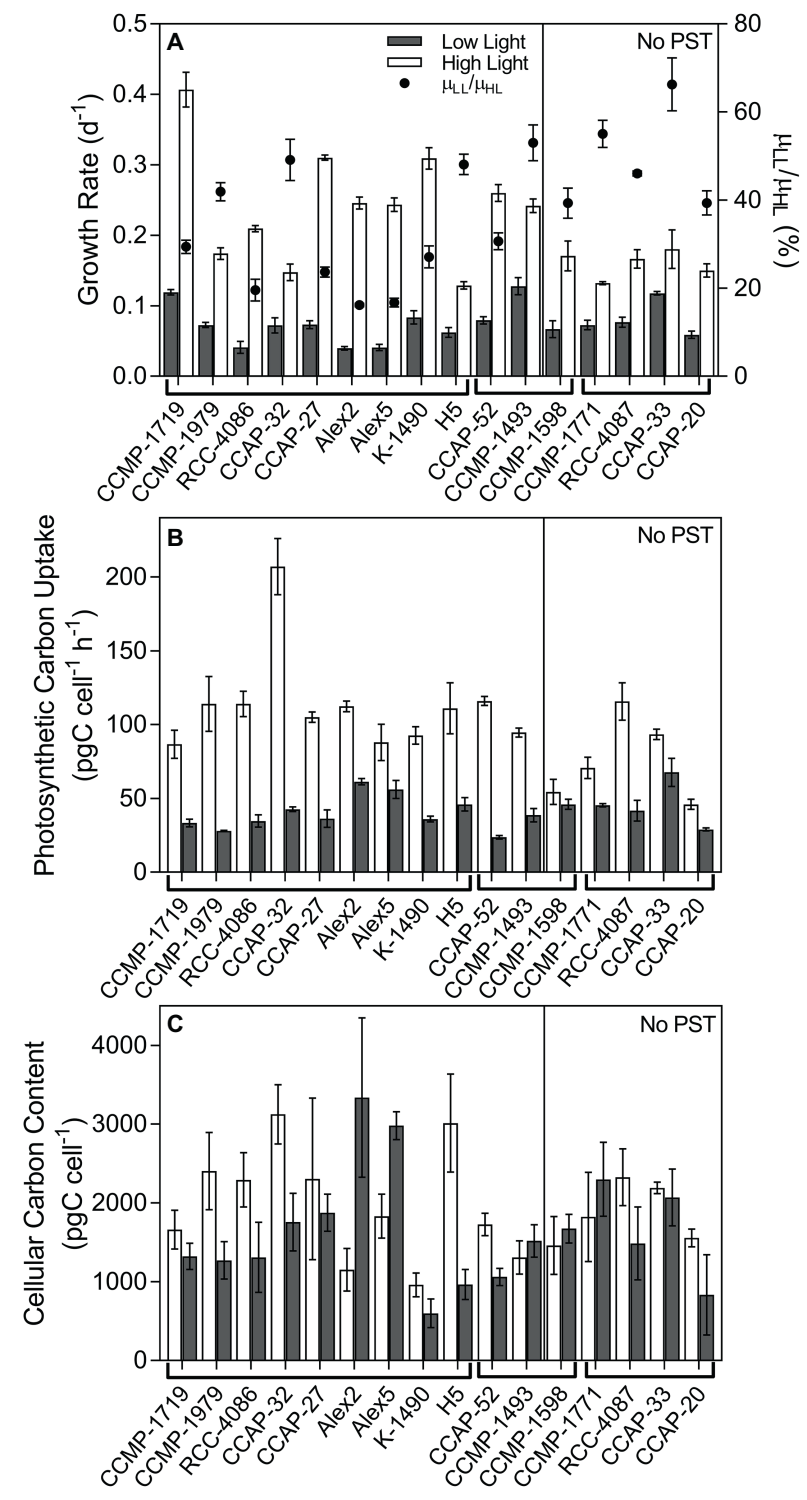
Growth, photosynthetic carbon uptake, and cellular carbon content of *Alexandrium* strains. **(A)** Specific growth rate of all strains (bars; left y-axis), and the growth at LL as a percentage of the growth at HL (μ_LL_/μ_HL_; black circles; right y-axis). **(B)** Photosynthetic carbon uptake (pgC cell^−1^ h^−1^). **(C)** Cellular carbon content (pgC cell^−1^). Strains are separated in two panels, the left side are PST-producers, and the right side are strains with no PST. The strains are separated further into species indicated just under the x-axis, the left nine strains are *A. catenella,* the middle three are *A. pacificum,* and the last four are *A. tamarense*. Strains grown in low light (LL; gray bars) and high light (HL; open bars). Error bars represent standard error, *N* = 3.

Growth rates varied among the selected strains from 0.04 to 0.13 and 0.13 to 0.41 d^−1^ in the LL and HL treatments, respectively, and the growth rates were reduced significantly in all strains in the LL treatment (Figure 1A, ANOVA with random effect of strain; *p* < 0.0001). The ratio of growth rates at LL compared to HL (=μ_LL_/μ_HL_), was used as an indication of how much the low light treatment had limited the specific algal culture by reducing the growth rate, compared to the growth rate at the high light treatment. In non-PST producers, the μ_LL_/μ_HL_ ratio was 0.50 ± 0.06 (mean ± SE, *n* = 5), and in PST producers, it was 0.32 ± 0.04, *n* = 11). Thus, the ratio of μ_LL_/μ_HL_ for the PST producers was significantly lower than the ratio of μ_LL_/μ_HL_ for the non-PST producers (*p* = 0.0211).

### Photosynthetic Carbon Uptake and Cellular Carbon Content

Photosynthetic carbon uptake varied among the strains but overall was significantly higher in HL than LL (*p* < 0.0001; Figure 1B), indicating the LL treatment limited carbon uptake. Cellular carbon content was highly variable between strains, and many of the strains were bigger under HL, but some were bigger under LL, while others did not differ in size whether grown at LL or HL. Overall, there was no significant difference between carbon content in cells grown at HL and LL (*p* = 0.0874; Figure 1C), and carbon content did not have a significant effect on the subsequent analyses. Most of the bacterial abundances made up <10% of the total particulate carbon measured. For a few of the strains, which had a lot of bacteria and/or were relatively small in size, the estimated carbon content of bacteria reached up to 25% of the total organic carbon in the culture (data not shown).

### Lytic Toxicity

**Figure 2:**
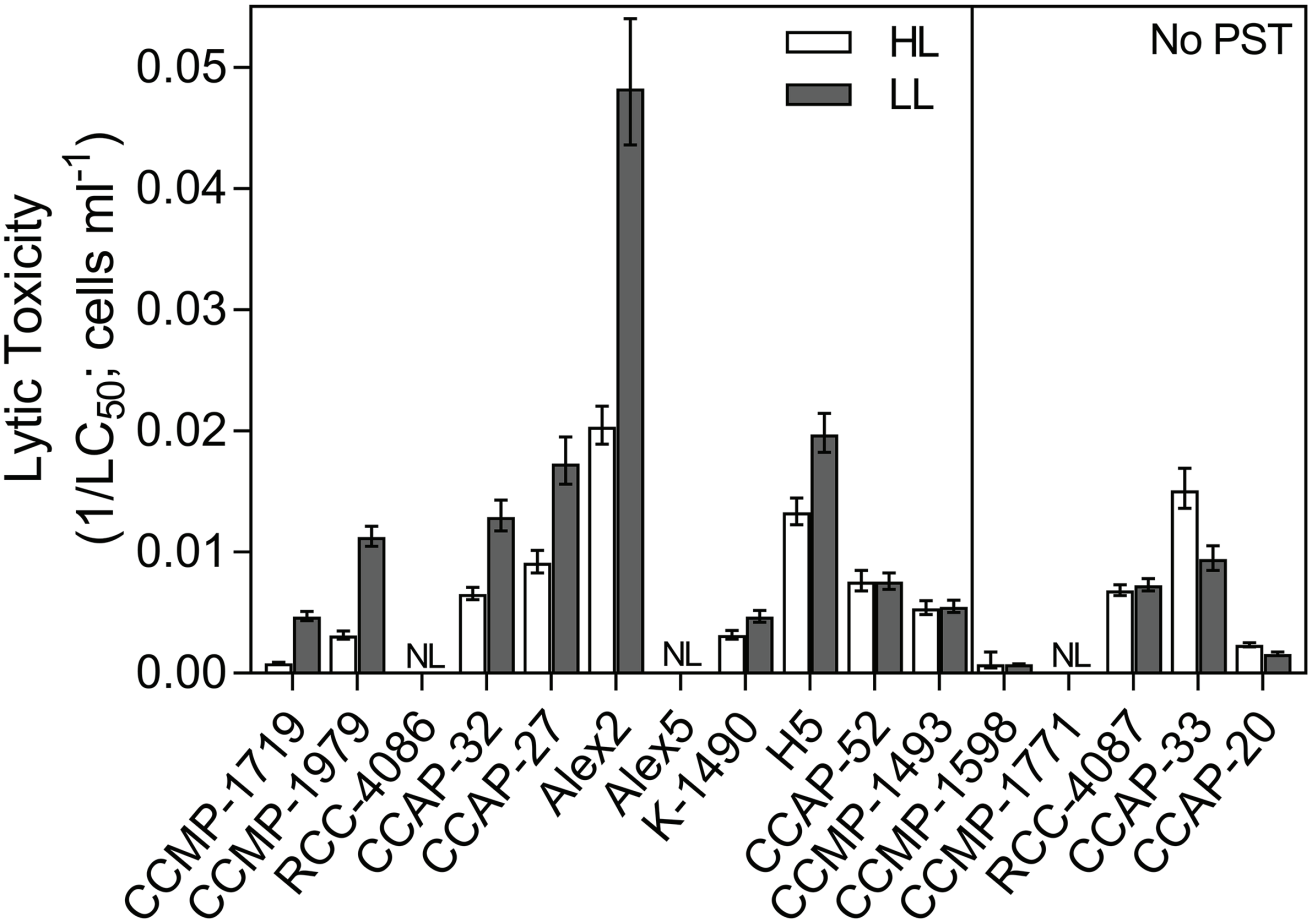
Lytic toxicity of all *Alexandrium* strains. Lytic toxicity was quantified as the concentration of *Alexandrium* causing 50% mortality of the target algae (LC_50_), calculated using a dose-response curve. Inverse LC_50_ (1/LC_50_) was used here to ease in the visual interpretation so that a higher number represents a higher lytic toxicity. Open bars represent strains grown in high light (HL) and grey bars represent strains grown in low light (LL). Non-PST producers are separated on the right panel. NL = non-lytic strains which did not reach densities high enough to cause any lytic effects. Error bars show 95% confidence intervals, *N* = 3.

Lytic toxicity was quantified as the concentration causing 50% cell lysis of *T. acuta* cells (LC_50_) and displayed as an inverse so that a higher number corresponds to a higher lytic toxicity (1/LC_50_; Figure 2). Only three out of the 16 *Alexandrium* strains had no measureable lytic effects at either light levels and could be considered non-lytic (Alex5, CCMP-1771 and RCC-4086). All other strains showed variation in lytic toxicity, with Alex2 being the most lytic with an LC_50_ of 21 cells ml^−1^ (Table 2; Figure 2). Overall, lytic toxicity was greater at LL than at HL, with a few exceptions. Because lytic toxicity is a measurement of the toxic effect in the medium, the compounds have been released and are assumed to be continually produced. This was confirmed by testing the stability of the lytic effect of the cell-free supernatant over time. The compounds lost bioactivity steadily and consistently whether at HL or LL (Supplementary Figure S1). For these reasons, lytic toxin “content” and “production” cannot be distinguished in the same way that they can be for the cell-bound and characterized PSTs.

**Table 2:** Results of growth and toxicity of all *Alexandrium* strains at high light (HL) and low light (LL).

Strain name	Species name	Growth rate (d^−1^ ± SEM)		Growth ratio (μ_LL_/μ_HL_)	PST content (pg cell^−1^ ± SEM)		Lytic toxicity LC_50_ (cells ml^−1^; 95% CI)	
		HL	LL		HL	LL	HL	LL
CCMP-1719	*A. catenella*	0.41 ± 0.01	0.12 ± 0.00	0.29	18.1 ± 1.26	12.6 ± 0.86	1,205 (1090–1,320)	213 (195–230)
CCMP-1979	*A. catenella*	0.17 ± 0.01	0.07 ± 0.00	0.43	40.7 ± 2.90	17.5 ± 1.40	321 (287–354)	89 (83–95)
RCC-4086	*A. catenella*	0.21 ± 0.00	0.04 ± 0.01	0.19	28.3 ± 1.65	11.2 ± 0.50	–	–
CCAP-32	*A. catenella*	0.15 ± 0.01	0.08 ± 0.01	0.47	83.8 ± 0.25	60.7 ± 3.19	153 (141–165)	77 (70–85)
CCAP-27	*A. catenella*	0.31 ± 0.00	0.07 ± 0.00	0.23	20.2 ± 1.81	13.4 ± 0.97	110 (98–121)	58 (51–64)
Alex2	*A. catenella*	0.25 ± 0.01	0.04 ± 0.00	0.16	5.8 ± 0.12	6.8 ± 0.31	49 (45–53)	21 (19–23)
Alex5	*A. catenella*	0.24 ± 0.01	0.04 ± 0.00	0.17	67.8 ± 3.57	32.1 ± 2.78	–	–
K-1490	*A. catenella*	0.31 ± 0.01	0.08 ± 0.01	0.26	11.2 ± 0.53	19.3 ± 0.84	318 (283–354)	215 (192–237)
H5	*A. catenella*	0.13 ± 0.00	0.06 ± 0.00	0.46	54.3 ± 5.20	31.8 ± 0.54	75 (69–81)	51 (47–55)
CCAP-52	*A. pacificum*	0.26 ± 0.01	0.08 ± 0.00	0.31	20.5 ± 1.52	9.7 ± 0.47	133 (118–147)	133 (121–144)
CCMP-1493	*A. pacificum*	0.24 ± 0.01	0.13 ± 0.01	0.54	27.0 ± 2.11	24.8 ± 0.63	187 (167–207)	182 (166–199)
CCMP-1598	*A. pacificum*	0.17 ± 0.01	0.07 ± 0.01	0.41	–	–	1,372 (573–2,172)	1,368 (1262–1,474)
CCMP-1771	*A. tamarense*	0.13 ± 0.00	0.07 ± 0.00	0.54	–	–	–	–
RCC-4087	*A. tamarense*	0.17 ± 0.01	0.08 ± 0.00	0.47	–	–	146 (137–156)	138 (128–147)
CCAP-33	*A. tamarense*	0.18 ± 0.03	0.12 ± 0.00	0.67	–	–	66 (59–73)	106 (95–118)
CCAP-20	*A. tamarense*	0.15 ± 0.01	0.06 ± 0.00	0.40	–	–	429 (396–462)	627 (576–678)

Specific growth rate (d^−1^), the ratio of growth rate at LL:HL (growth rate reduction), total cellular PST content (pg cell^−1^), and lytic toxicity as LC_50_ (Alexandrium cells ml^–1^).

“–”, no PST detected or no measureable lytic effects.

### PSTs

**Figure 3:**
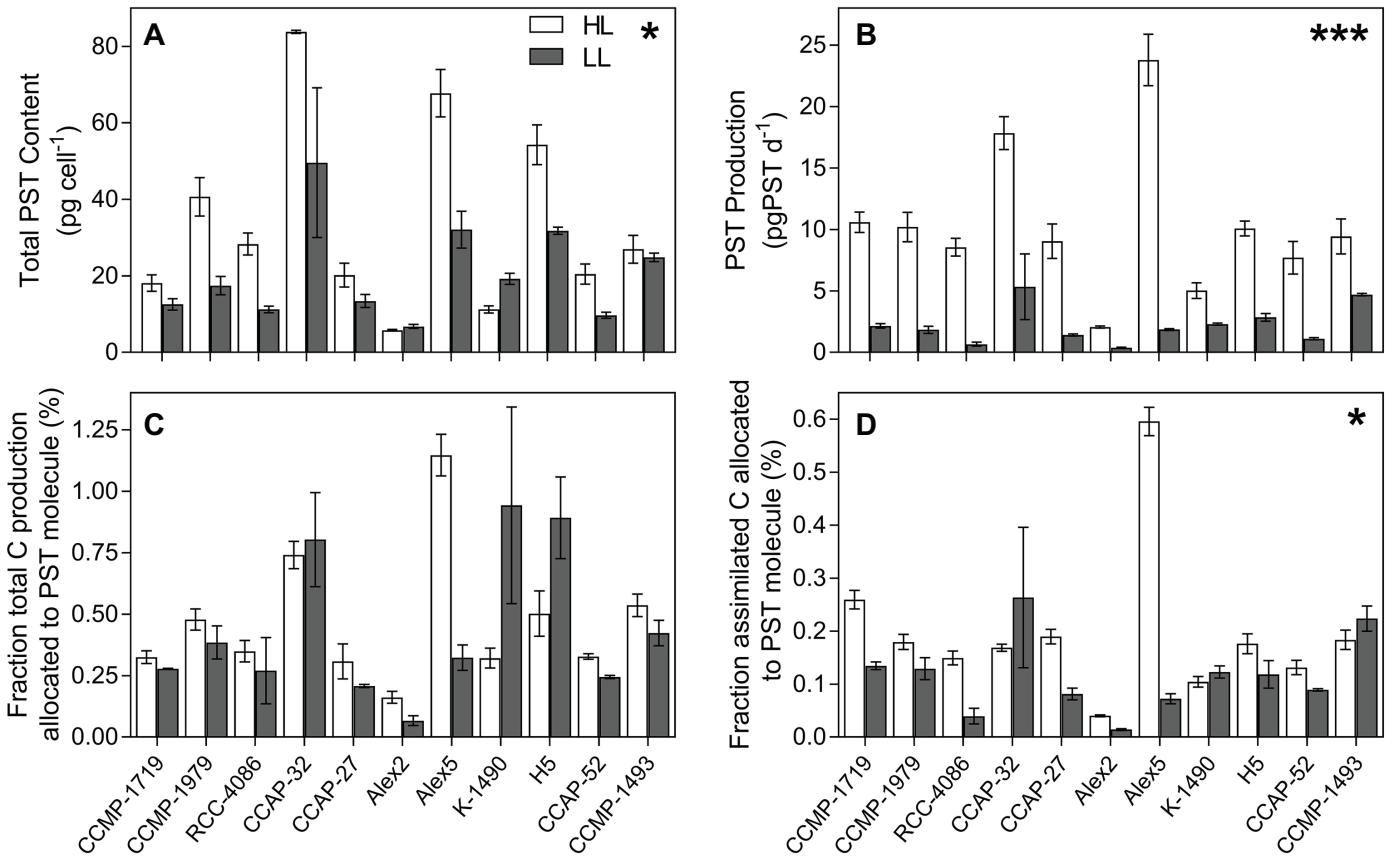
PST content and production. **(A)** Total PST content (pg cell^−1^). **(B)** PST production (pg cell^−1^ d^−1^). **(C)** PST production in proportion to total production of each strain shown as the percentage C in the PST molecule compared to the total cellular C content indicating how much C produced is allocated towards production of the PST molecule. **(D)** Percent C assimilated through photosynthesis that is allocated towards the PST molecule. For **(C,D)** the C needed for PST synthesis and metabolism is not included in the C allocated towards PST (see Supplementary Materials for estimates including synthesis costs; Supplementary Figure S3). Open bars represent strains grown in high light (HL) and grey bars represent strains grown in low light (LL). Error bars represent standard error; *N* = 3. * indicates level of significance, * <0.05, *** <0.0001 (HL significantly greater than LL in all cases, except **(C)** which had no significant difference between light level).

Eleven out of the 16 strains analyzed contained PSTs (Table 2). The total PST content per cell was calculated combining the six analogs, C1/C2, GTX1/4, GTX2/3, B1, NEO, and STX. Overall, PST content was significantly greater when strains were at HL, despite three exceptional strains (*p* = 0.013; Figure 3A). All strains had significantly greater PST production rates in HL (*p* < 0.0001; Figure 3B), but because this could simply be due to the higher growth rates at HL, the PST production was normalized to total production as well as to total assimilated carbon (Figures 3C,D). These calculations only included carbon in the PST molecules, not carbon used for toxin synthesis and metabolism. Overall, normalizing PST production to total production decreased the differences in most of the strains between toxicity at HL vs. LL, and there was no significant difference between percent carbon production allocated to the PST molecule (*p* = 0.353; Figure 3C). In HL, Alex2 and Alex5 are in the extremes, with over 1% of the cellular carbon content allocated toward PST in Alex5, and with PST making up less than 0.14% cellular carbon content of Alex2, and nearly half of that at LL (Figure 3C). In terms of allocation costs, the majority of the strains allocate more assimilated carbon toward PST production in the unlimited high light conditions (*p* = 0.025; Figure 3D). Estimates of allocation costs including carbon used for toxin synthesis and metabolism were done using calculations by Chakraborty et al. (2018), and references therein (Supplementary Figure S3).

All the strains identified as *A. catenella* produced PST, and all the strains identified as *A. tamarense* lacked PST. Two out of the three *A. pacificum* strains produced PST (CCMP-1493 and CCAP-52), and one did not (CCMP-1598). Because this was a trait-based analysis (the traits being lytic toxin production and PST production), rather than a comparison between species, we split the strains into PST producers and non-PST producers, and lytic or non-lytic strains. Because all *A. catenella* strains produced PST and all *A. tamarense* strains lacked PST, there is a confounding effect between species and PST presence/absence, making it impossible to accurately interpret results if both factors are considered in the same statistical analysis. Because we wanted to include lytic toxicity and PST in the same statistical model, and the majority of all species were lytic, we determined that the best way to explain the data would be to split it up into PST and non-PST producers, rather than species.

### Trade-Off Between Growth, PST and Lytic Toxicity

**Figure 4:**
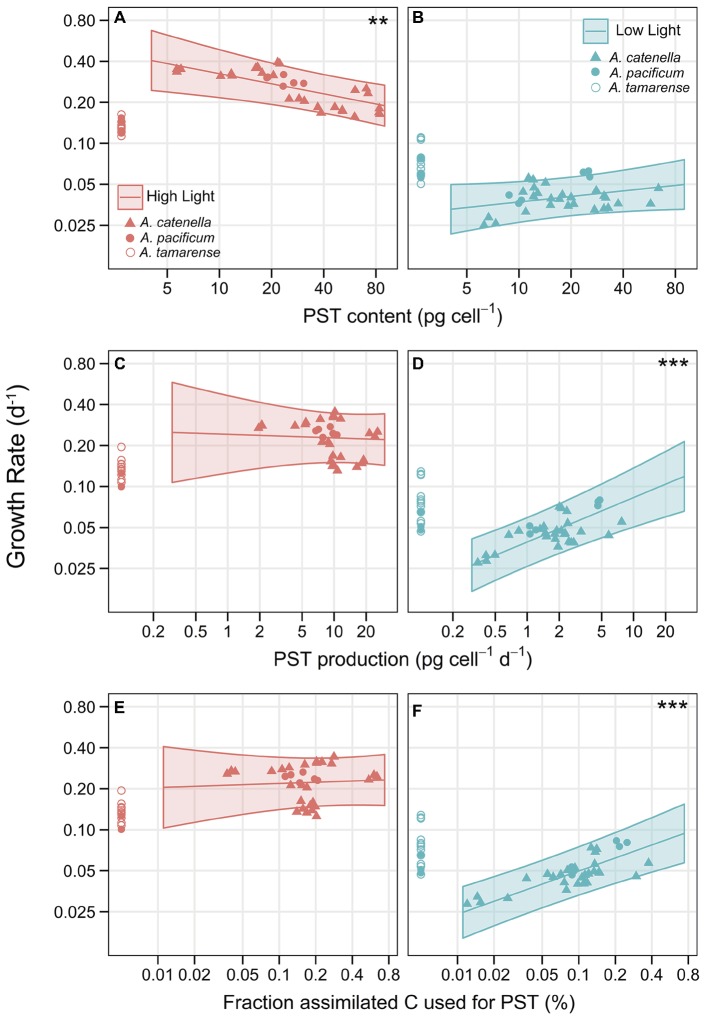
Trade-off curves of PST with lytic toxicity on growth. Effect of PST on growth with PST expressed as **(A)** PST content (pg cell^−1^) at HL and **(B)** at LL. **(C)** PST production (pg cell^−1^ d^−1^) at HL and **(D)** at LL. **(E)** Proportion of assimilated C allocated towards the PST molecule (%; excluding C needed for synthesis), at HL and **(F)** at LL. Left panels (in red) are in HL conditions and right panels (in blue) are LL. The only negative effect of PST on growth, indicating a cost, was evident under HL when PST is expressed as PST content (A, *p* = 0.0039). There was a significant, positive effect of PST on growth under LL, when considering either PST production (D, *p* < 0.0001), or the proportion assimilated C allocated towards the PST molecule (F, *p* < 0.0001). Symbols show observed growth rates corrected for lytic toxicity according to the ANCOVA models and denote species: *A. catenella* (triangles), *A. pacificum* (closed circles), and *A. tamarense* (open circles); non-PST producing strains were placed close to the y-axis. Each triplicate of the 16 strains are represented as a symbol. For a corresponding figure displaying the observed growth only, without a correction for lytic toxicity, see Supplementary Figure S2. The lines show ANCOVA model predictions for PST producing, non-lytic strains with the shaded areas indicating 95% CI. * indicates level of significance of the slope, ** < 0.01, *** < 0.001. Axes are on a log–log scale.

An analysis of covariance (ANCOVA) with random effects of strain, and light treatment nested within strain, was done to investigate the effect of PST and lytic toxicity on growth. Cellular carbon content (biomass) was initially included as a possible covariate but was removed because analyses showed it did not explain the growth response (see Methods). To avoid overlooking any potential evidence of costs, PST was expressed in three ways, PST content, PST production, and the fraction of assimilated carbon (C) used to produce the PST compound. An ANCOVA analysis was done for each expression of PST. All three analyses showed highly significant interactions between PST and light (*p* = 0.0001, *p* = 0.0002, *p* = 0.0009, for PST content, production, and C fraction, respectively), a significant interaction between lytic toxicity and whether the strains contained PST (*p* = 0.0157 for PST content), and significant effects of lytic toxicity on growth (*p* = 0.0125, *p* = 0.0175, for PST production and C fraction, respectively). For PST, evidence of a cost, in terms of growth reduction with increasing toxicities, was only apparent for PST content at HL, where there was a negative effect of PST content on growth (Figure 4A, *p* = 0.0039). The opposite was found at LL, where there was a positive effect of PST on growth that was significant when considering either PST production (Figure 4D; *p* < 0.0001) or fraction of assimilated C used for PST (Figure 4F; *p* < 0.0001). Therefore, under C limitation, there was no evidence of a cost to PST (Figures 4B,D,F). Because lytic toxicity and PST are combined in one statistical model, the effects of lytic toxicity on growth are also combined with PST effects visually and displayed in Figure 4 by correcting the observed growth of each strain for lytic toxicity (see Supplementary Figure S2 for visualization of effects of PST on growth without correcting for lytic toxicity).

**Figure 5:**
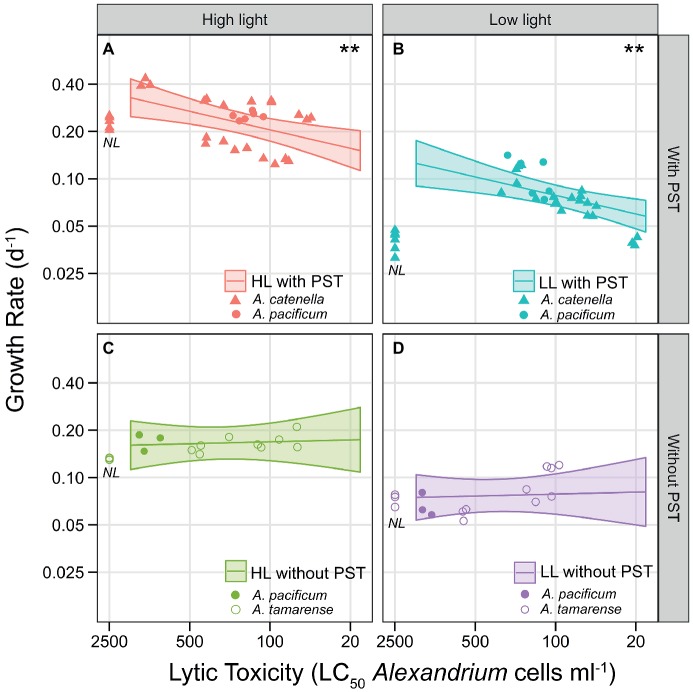
Trade-off curves of lytic toxicity, showing the interaction with PST presence/absence. Lines show ANCOVA model predictions at the average PST level (geometric means computed within each panel), with shaded area representing point wise 95% confidence intervals. Symbols are the observed values and denote species, *A. catenella* (triangles), *A. pacificum* (closed circles), and *A. tamarense* (open circles). Each triplicate of the 16 strains are represented as a symbol. Non-lytic strains are placed near the y-axis (NL). **(A)** HL PST producers, **(B)** LL PST producers, **(C)** HL without PST (non-PST producers), **(D)** LL without PST (non-PST producers). Both axes are on a log-scale. The x-axis shows lytic toxicity as inverse LC_50_ (1/LC_50_), but is labeled with the actual LC_50_ cell concentrations (cells ml^−1^), so lytic toxicity increases along the x-axis. ** indicates significant slopes (in this case, *p* = 0.0019), only found here in the PST producers **(A,B)**.

To isolate the effects of lytic toxicity on growth to reveal the costs, Figure 5 shows the observed growth correlated with lytic toxicity but separated between PST producing strains and non-PST producers. Overall, there was a significant negative effect of lytic toxicity on growth when using PST production or C fraction of PST to express PST (*p* = 0.0125 and *p* = 0.0175, respectively). When expressing PST as content, there was a negative effect of lytic toxicity, but only for PST-producing strains (Figures 5A,B; *p* = 0.0019) and no significant effect for non-PST producing strains (Figures 5C,D; *p* = 0.8083). Therefore, we chose to display the effects of lytic toxicity on growth including this interaction in Figure 5. Thus, the significant cost of lytic toxicity was only evident for PST producers (Figures 5A,B compared to Figures 5C,D). The conservative model selection method resulted in the same slope for HL and LL, both significant, but visually the observed slope may be steeper at LL when C was limited.

Supplementary Figure S5 shows the predicted average growth rates in eight regimes defined by light (HL/LL), PST production (yes/no), and lytic toxicity (yes/no), and the highest growth was found under HL for PST-producing strains (Supplementary Figure S5). The measurement of the strains in triplicates at both light conditions allows for comparison of the biological and the experimental variation. When expressing PST as production or fraction, 64% of the variation may be attributed to variations between strains, and 29% was due to variation between light treatments within strains, while only 7% was due to variation between triplicates (Supplementary Tables S2, S3). See Supplementary Tables S1–S3 for all parameter estimates and variance components.

## Discussion

### Costs of Lytic Toxicity

We found measurable intrinsic growth rate costs of lytic toxin production in *Alexandrium*, providing the first empirical evidence of significant lytic toxin production costs. These costs were measureable as reduced growth rates correlated with increasing lytic toxicity but only in the strains that produced PST (Figures 5A,B). The trade-off of lytic toxicity with growth was more pronounced at LL than at HL (LL compared to HL; Figures 5A,B), indicating costs are inflated at low light when strains were subject to carbon limitation. Surprisingly, most strains were more lytic at LL than at HL (Figure 3). This was not an artifact of light sensitivity of the compounds, or dilution of the observed effect due to faster cell division at HL because the biological activity of the lytic compounds declined within hours and at nearly the same rate in both HL and LL (Supplementary Figure S1). Therefore, the higher lytic toxicities at LL reflect maintenance of lytic toxin production even as photosynthesis is reduced and indicate LL conditions are where the ecological function provides a benefit such as using lytic compounds to capture prey, or possibly predation defense.

Consistent with other studies (Tillmann and John, 2002; Fistarol et al., 2004), there was no significant relationship between the strength of lytic effects and the amount of PSTs at the two light levels. Despite this, when expressing PST as content, there is a highly significant relationship between the presence of PST and the growth trade-offs of lytic toxicity (Figure 5). Furthermore, the PST producers are the most lytic strains at low light. Under carbon-unlimited conditions (HL), the PST-producing strains and the non-PST-producing strains did not differ as much in their lytic toxicities (Figures 5A,C). This interaction effect could explain previously reported inconsistencies in the effects of *Alexandrium* spp. on predation, which typically only report PST toxicity. Although occasionally mentioned, the lytic compounds have largely been overlooked in many of the predator defense studies using copepods (Ianora et al., 2012; Xu and Kiørboe, 2018), but it is now obvious that PST producers gain something from the production of lytic compounds. The potential for lytic compounds to have a role in grazer defense, as opposed to the PSTs (Xu and Kiørboe, 2018), perhaps as a signal to deter grazers, deserves further attention. Considering the now proven cost of lytic toxicity, we would have expected the non-lytic strains in our experiments to grow faster than the other strains. Interestingly, the non-lytic strains (NL in Figure 5), actually have comparable growth to the model predictions for lytic algae in all cases except for when costs become most apparent (LL). Under low light conditions, the PST-producing strains that are most lytic grow faster than, or the same as, the non-lytic strains (Figure 5B). In this case, the non-lytic strains are growing far lower than “predicted” as they should grow faster than even the least lytic strains. If the non-lytic strains are in fact receiving a benefit, they do not appear to have increased fitness when costs are significant.

Although rare (Alpermann et al., 2010), the existence of non-lytic strains, or “cheaters” that receive the shared benefits of lytic compounds, without suffering the associated costs, has led some to suggest that the release of lytic compounds used in competitive interactions (allelopathy) may not be an adaptive trait (Lewis, 1986; Jonsson et al., 2009). The potential for “cheaters” and the threshold dependency of lytic effects raises important questions about the level of selection (i.e., individual, population, species), as well as the scale of the benefits and the potential for altruism (Driscoll et al., 2013, 2016; John et al., 2015). If lytic toxicity was exclusively altruistic, the non-lytic strains would have increased fitness compared to their lytic counterparts and should increase in frequency, undermining the public good. Ultimately, this leads to the tragedy of the commons (Hardin, 1968; Driscoll et al., 2016), weakening the stability of lytic toxicity as an adaptive trait selected for competitive inhibition *only* after threshold concentrations are reached. However, an altruistic trait may persist and avoid the tragedy of the commons if individual benefits also exist, with some level or periods of exclusivity to the producer (Driscoll et al., 2016).

Evidence of toxin-assisted mixotrophy provides the most convincing support for cell-level benefits of toxins that are released from the cells into the surrounding water. Lytic compounds have been suggested to aid in prey capture of motile prey in *Prymnesium parvum* (Skovgaard and Hansen, 2003; Tillmann, 2003), *Karlodinium armiger* (Berge et al., 2012) and *Karlodinium veneficum* (Adolf et al., 2006; Sheng et al., 2010). In these cases, lytic compounds are released into the surrounding medium. In other cases, mucus is also released perhaps as a way to physically stabilize the lytic compounds in *Dinophysis* spp., (Mafra et al., 2016; Ojamäe et al., 2016; Papiol et al., 2016) and *A. pseudogonyaulax* (Blossom et al., 2012).

Cell-level benefits may be difficult to measure, and it is the more conspicuous community level benefits that we are able to observe as lytic effects. The relatively high cell concentrations that are often required to elicit allelopathic effects in the laboratory are often only found in nature after blooms have been established. This was the premise for Jonsson et al. (2009) to suggest that lytic compounds used as chemical inhibition of competing protists cannot be responsible for the initiation of HABs. However, we show that lytic effects can be observed at very low cell concentrations (the most lytic strain had an LC_50_ of just 21 cells ml^−1^). In fact, the LC_50_s of the most lytic strains shown here, particularly the PST producers, would have been considered “low,” pre-bloom concentrations in the meta-analysis described in Jonsson et al. (2009). The threshold concentrations would be even lower, meaning lytic effects relevant to the *Alexandrium* cells may not require high concentrations (or a collective effort) of lytic cells. Although the target algae used here is quite sensitive, the reported effects are with cell-free supernatant, and after only 3 h of exposure, so the effects in nature are likely occurring at even lower cell densities.

### Costs of PST

There was no clear evidence of a measureable fitness cost to PST under carbon limitation; in fact, PST had a significant positive effect on growth in LL (Figures 4D,F). Costs in terms of growth rate reduction were only evident with cellular PST content in unlimited, HL conditions. Previous studies have not been able to show measureable costs to PST production. For example, cells which induce PST production when copepods are present grow just as fast as un-induced strains (Bergkvist et al., 2008). Previous model simulations by John and Flynn (2002) predict that PST costs are negligible and suggest they may be “selection neutral”, neither selected for or against.

A grazer defense mechanism has been the leading explanation for the evolution of algal toxins, and PST specifically (Turner and Tester, 1997; Smetacek, 2001). This concept was initially inspired because of the neurotoxic nature of these compounds and has persisted mainly because grazer cues increase PST content in some strains (Selander et al., 2006; Bergkvist et al., 2008; Senft-Batoh et al., 2015). Recently, the defensive role of PSTs was modeled in *Alexandrium,* predicting that costs are only measurable at low nitrogen concentrations and when grazer biomass is high (Chakraborty et al., 2018). However, the C required for PST synthesis, in addition to C used in the PST molecule, suggests C limitation, as used here by reducing irradiance, should reveal direct measureable reductions in growth rate, despite nutrient replete conditions. However, under C limitation, no cost could be measured (LL; Figures 4B,D,F); rather PST production (and fraction assimilated C) was positively correlated to growth (Figures 4D,F). Carbon makes up between 25 and 40% of the total molecular weight of the saxitoxin analogs, which means that PSTs measured in these experiments make up ~1% or less of the total carbon content of the *Alexandrium* cells. Applying the model estimates of biosynthesis costs of PST to our strains (using calculations done by Chakraborty et al., 2018 and references therein), investment in PST production does not correlate with growth reduction, at either HL or LL (Supplementary Figure S4).

In addition to the lack of fit of our data to the model predictions of a defensive role of PST (Supplementary Figure S4), the significant relationships between growth, PST, and light found in our study, *despite* the lack of selection pressures such as grazers or nitrogen limitation, suggest an alternative or additional role of PST should not be ignored. The elevated growth rates of PST producers compared to non-PST producers at HL (symbols displayed near the y-axes in Figure 4; Supplementary Figure S5), the apparent cost of PST at HL rather than random scatter and the unexpected benefit (rather than cost or random scatter) of PST during C limitation, all support that a function of PST in internal cellular processes may be just as plausible as predation defense. The potential role of PST in photosynthesis, non-photochemical quenching of photosystem II, or C acquisition deserves further investigation.

Aside from predator defense, there have been limited additional hypotheses tested for an alternative role of algal toxins. Although brevetoxins produced by the dinoflagellate *Karenia brevis* have also been suggested as a predator defense (Hong et al., 2012; Hardison et al., 2013), they have recently been implicated for involvement in photosystem II (Cassell et al., 2015). Glibert et al. (2016) proposed that toxin production in HABs could be related to photosynthesis and pathways of nitrogen and carbon metabolism, which may also be influenced by the nitrogen source (ammonium/urea/nitrate). The toxins of *K. veneficum* are only produced during the light period (Adolf et al., 2008), suggesting a link to photosynthesis (Glibert et al., 2016). Often toxicity in the field is far higher than what can be found in the lab (Hardison et al., 2013; Brosnahan, personal communication), meaning nutrient limitation, higher irradiances, presence of predators and prey as well as a number of different environmental factors may be important for a deeper understanding of the ecological role of algal toxins.

### Species Designations

This study was a trait-based approach to investigating the costs of toxin production in one of the most prominent HAB genera, *Alexandrium.* Therefore, our strain selection, data analyses and interpretation of the results did not focus on what the species designations were, but rather what the toxicity of the strains were. Nevertheless, proper identification is essential, and it turned out that all *A. catenella* strains were PST producers, all *A. tamarense* strains did not produce PST, whereas strains of *A. pacificum* produced both; all three species had lytic strains. All other physiological parameters measured here (cellular carbon content, carbon uptake, carbon production, lytic toxicity, and growth rate) were not significantly different between species at either high or low light (ANOVA, data not shown). Thus, although it is difficult to separate PST presence/absence from the difference between *A. catenella* and *A. tamarense*, in the data set here, we suggest PST presence/absence (as opposed to species designation) as the defining physiological characteristic of these strains in regards to the cost of toxin production.

### Strain Variability

Like previous studies, (Tillmann et al., 2009; Alpermann et al., 2010; Blossom et al., 2012; John et al., 2015), there was large variation among strains in terms of toxicity, but also in terms of C uptake, growth rates, cellular C content, and even bacterial abundances. There was even variation in how a strain responded to irradiance treatments. Growth and photosynthetic activity were the only two parameters that were consistently higher in all strains grown in HL compared to LL. The other parameters (cellular C content, lytic toxicity, PST content) had a trend to be either higher in LL or in HL, but there was always exceptional strains, which were rarely the same strains across all parameters. The fact that correlations are revealed despite this variation (up to 64% of all variance is due to strain compared to 29% due to treatment; Supplementary Tables S2, S3) gives great confidence to the trends found here. The use of enough strains and interpretation of the results using a random-effects model sets our study apart from many previously done and could be part of the explanation of why previous studies show contradictory results, particularly in the role of toxicity for grazing defense (Waggett et al., 2012; Turner, 2014; Xu et al., 2017).

## Conclusions

For the first time, we demonstrated measureable growth rate costs of toxin production, under certain environmental conditions. Particularly, the fitness costs of lytic toxicity for PST producers are not trivial. The costs of PST production on growth were surprisingly only apparent at high light, where strains containing PST also grew faster than non-PST producers. PST production was positively correlated with growth under light limitation, and the role of PST in carbon acquisition and photosynthesis requires further study. Trade-off curves provided here can be used to model toxicity in relation to environmental conditions and it is now important to expand beyond fitness costs to ecological costs and how these costs compare to the benefits found in nature, with competitors, grazers, and prey. This will help us move forward in determining the ecological function and evolutionary relevance of toxin production in microalgae.

## Author Contributions

HB designed, planned, and performed the experiments, collected and analyzed the data, and wrote the manuscript. PH designed and planned the experiments, assisted with data analysis and writing of the manuscript. BM assisted with data analysis, guided, and performed most of the statistical analysis, wrote sections of the manuscript, and edited the manuscript. ND performed species identifications, wrote sections of the manuscript, and edited the manuscript. BK quantified PSTs and edited the manuscript. AN performed bacterial measurements on flow cytometry and edited the manuscript.

### Conflict of Interest Statement

The authors declare that the research was conducted in the absence of any commercial or financial relationships that could be construed as a potential conflict of interest.
