# Supplementary material for: The Cost of Toxicity in Microalgae: Direct Evidence From the Dinoflagellate Alexandrium
Source: Front Microbiol. 2019 May 22;10:1065. doi: 10.3389/fmicb.2019.01065 (PMC6538772; doi:10.3389/fmicb.2019.01065)
Supplement: Supplementary file 1 [file Data_Sheet_1.docx]

Supplementary Material

The cost of toxicity in microalgae: direct evidence from the dinoflagellate *Alexandrium*

Hannah E. Blossom*, Bo Markussen, Niels Daugbjerg, Bernd Krock, Andreas Norlin, Per Juel Hansen

*** Correspondence:** Hannah E. Blossom: [blossom.hannah@gmail.com](mailto:blossom.hannah@gmail.com); hblossom@bio.ku.dk

# Supplementary Text

**Species identification**

**Methods.** Approx. 10 mL of all 16 strains of *Alexandrium* were harvested by centrifugation (15 min at 3000 x *g*, 15 °C), and cell pellets were subsequently frozen at -18 °C for further analysis. Cell pellets were thawed and extraction of total genomic DNA was made using the Powerplant Pro DNA Isolation kit (MO BIO Laboratories Inc., Carlsbad, CA, USA). The procedure followed the recommendations of the manufacturer. PCR amplification of approx. 1500 base pairs of the nuclear-encoded large subunit ribosomal DNA gene (LSU rDNA) was done with the forward primer D1F (Scholin et al., 1994) and the reverse primer 28-1483R (Daugbjerg et al., 2000). Amplification conditions were one initial cycle of denaturation at 95 °C for 12 min followed by 35 cycles each consisting of denaturation at 95 °C for 1 min, annealing at 59 °C for 1 min and extension at 72 °C for 3 min. The final step included extension at 72 °C for 5 min. The amplification kit used was 5X Hot FIREPol Blend Master Mix from Solis BioDyne (Tartu, Estonia). To confirm the expected length of the PCR products 4 µL of the PCR amplified products were run in 1.5% agarose-casted gels for 9 min at 130 V. Amplified products were stained using GelRed and visualized using a gel documentations XR System from BioRad (Hercules, CA, USA). Fragment length was compared to a 100-base-pair RAIN-BOW eXtended DNA ladder (BIORON GmbH, Ludwigshafen, Germany). PCR products were purified using ultrafiltration with the Nucleofast 96 PCR kit from Macherey-Nagel (GmbH & Co, KG, Düren, Germany) following the manufacturers recommendations. DNA concentrations were measured using a Nanophotometer P300 (Implen, GmbH, München, Germany).

LSU rDNA sequences were determined in both directions using the amplification primers in addition to the internal primer D2C-R (Scholin et al., 1994). The service provided by Macrogen (Amsterdam, Netherlands) was used for sequence determinations. CLC Main Workbench (ver. 7, Qiagen Bioinformatics, Århus, Denmark) was used to trim and assemble all sequences. Contigs were obtained with default settings.

**Bacterial abundance**

**Methods.** Subsamples for bacterial abundance were taken continually during the exponential growth of the strains at the same time as subsamples for cell counting. 960 µL of culture was put into a 2 mL Eppendorf tube, and fixed with 1% final concentration of glutaraldehyde. These were placed in 4 °C for at least 2 hours in order for proper fixation, and then were stored in -80 °C until further analysis. Bacterial abundance at the beginning, middle and end of the exponential growth for each strain were determined using a FACS Canto II flow cytometer (BD Biosciences). The sample from mid-exponential phase was selected for each strain from the same day or in a few cases at LL, within 3 days, of the TOC and photosynthesis measurements for that particular strain. Carbon content of the bacteria was estimated as 20 fg C cell^-1^ (Lee and Fuhrman 1987), and this was used to calculate an estimated fraction of total particulate organic carbon made up of bacteria. This was subtracted from the POC calculated to get more accurate algal cellular carbon contents.

**Lytic toxicity degradation in light**

**Methods.** Preliminary results suggested elevated lytic toxicities under LL compared to HL, leading to the question of stability of the lytic compounds, and if light had an influence on how fast the lytic compounds were degraded. If the compounds degraded much faster at HL, higher lytic toxicity at LL would not necessarily mean more lytic compounds were released, but rather they were more stable, and lytic effects stronger. Therefore, it was deemed necessary to test if there was a major difference in degradation of the lytic compounds in high light and low light using the two most lytic *Alexandrium* strains. One liter of both H5 and Alex2 were grown in one glass Pyrex® bottle at high light. During exponential phase, the culture was first diluted to a relevant cell concentration, that is one that would have been used and causing toxicity in the experiments performed in this study, which was approximately 500 cell mL^-1^. This culture was then centrifuged at 3000 x *g* for 15 minutes. The cell-free supernatant was retained and pooled for each strain, and then separated into triplicate 100 mL blue-capped bottles, with 66 mL volume of supernatant in each bottle. One set of triplicates was placed at HL, and the other at LL. At this time, a lytic toxicity test was performed in order to obtain an initial lytic toxicity estimate. After 6 hours, a 10 mL aliquot from each triplicate was taken for a complete dose-response curve. This was repeated after 21 hours and again after 48 hours. Unfortunately, for Alex2 in high light, the samples were compromised and could not be used for testing. The test was performed as stated above following the *T. acuta* microalgal bioassay to estimate an LC_50_, and track the degradation of the lytic compounds over time.

**Results.** A lytic toxicity test over time was done on two of the most toxic strains (H5 and Alex2) to determine if the lytic effects seen in the LL treatment were stronger than in the HL treatment due to slower degradation of the lytic compounds in LL than in HL treatments. The supernatant of two of the toxic strains placed at LL and HL started losing lytic effects if they were left for just 6 hours prior to the lytic test, but the supernatants were still equally lytic whether they were placed at LL or HL (Figure S1). A slight difference was seen between HL and LL treatments if the supernatants were left for 21 hours prior to measuring lytic activity for both strains. However, the lytic toxicity was not strong enough to cause greater than 30% mortality for Alex2, and 20% mortality for H5. The lytic toxicity of Alex2 supernatant kept at LL for 21 hours prior to measuring lytic activity was slightly higher than the corresponding supernatant kept at HL (Figure S1); this difference however was not nearly as large as the difference between lytic toxicities of the cultures grown at LL and HL (Figure 3), and thus does not influence the interpretation of the results. After 48 hours nearly all toxicity was lost for both strains regardless of light level, and no LC_50_ could be estimated.

# Supplementary Figures and Tables

## Supplementary Figures

**Figure S1.** Degradation of released lytic compounds in the supernatant of strains Alex2 and H5 under culture conditions. Grey bars are the initial lytic toxicity as expressed by inverse LC_50_, open bars are after storage under HL and closed bars are after storage under LL. Error bars show 95% confident intervals. At 48 hours, both strains at both light levels (no data for Alex2 at HL) did not cause enough mortality to calculate an estimated LC_50_.


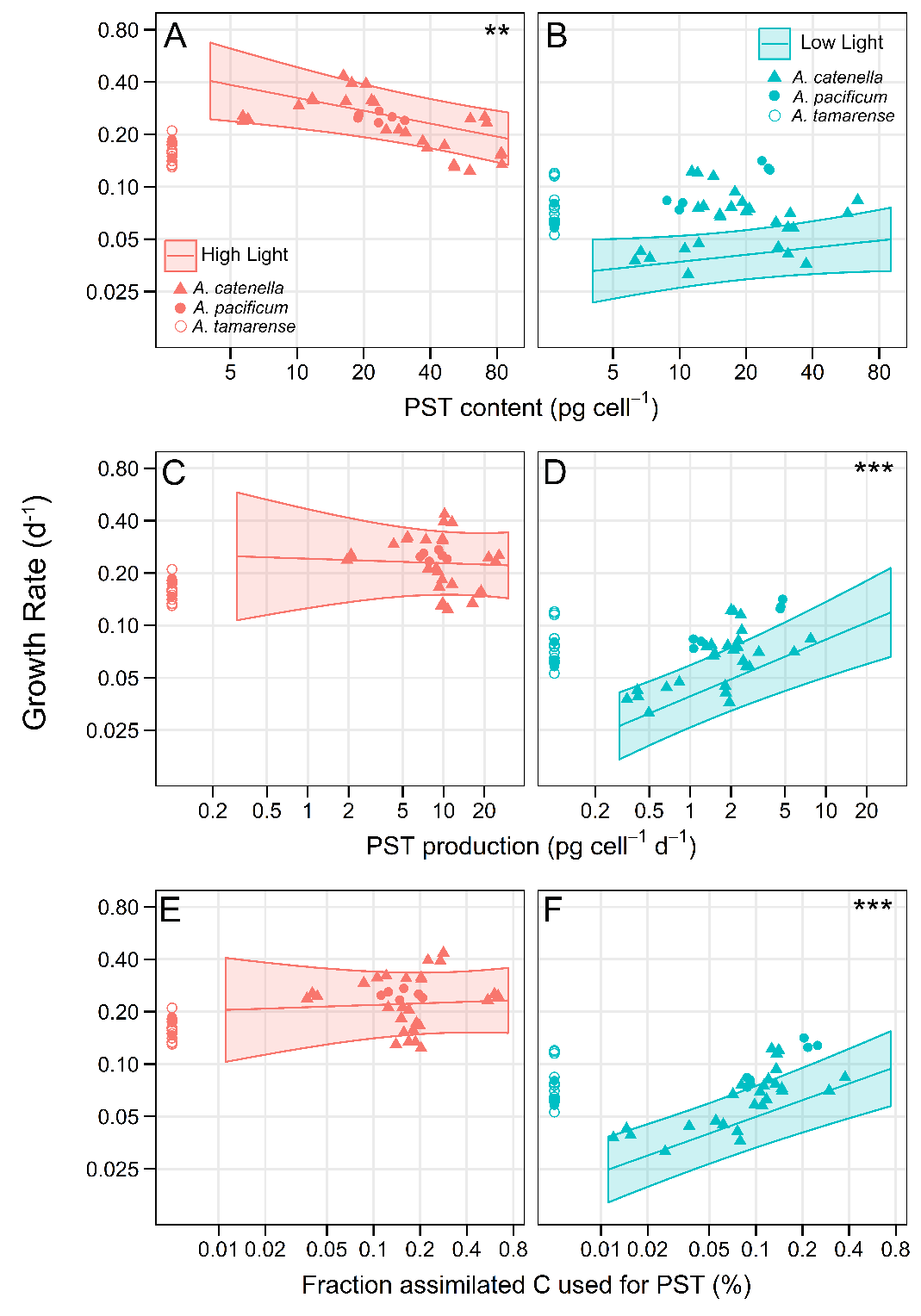


**Fig. S2.** Effect of PST on growth, showing observed growth. PST is expressed as **(A-B)** PST content (pg cell^-1^), **(C-D)** PST production (pg cell^-1^ d^-1^), and **(E-F)** proportion of assimilated C allocated towards the PST molecule (%; excluding C needed for synthesis). Left panels (in red) are in HL and right panels (in blue) are LL. Symbolsshow observed growth, before correcting for lytic toxicity, as opposed to the corrected growth shown in Fig. 4. In this way, we can see how the observed growth shifts after correcting for lytic toxicity for a well-fitting statistical model (line with shaded area showing 95% confidence intervals). Symbols denote species: *A. catenella* (triangles), *A. pacificum* (closed circles), and *A. tamarense* (open circles); non-PST producing strains were placed close to the y-axis.


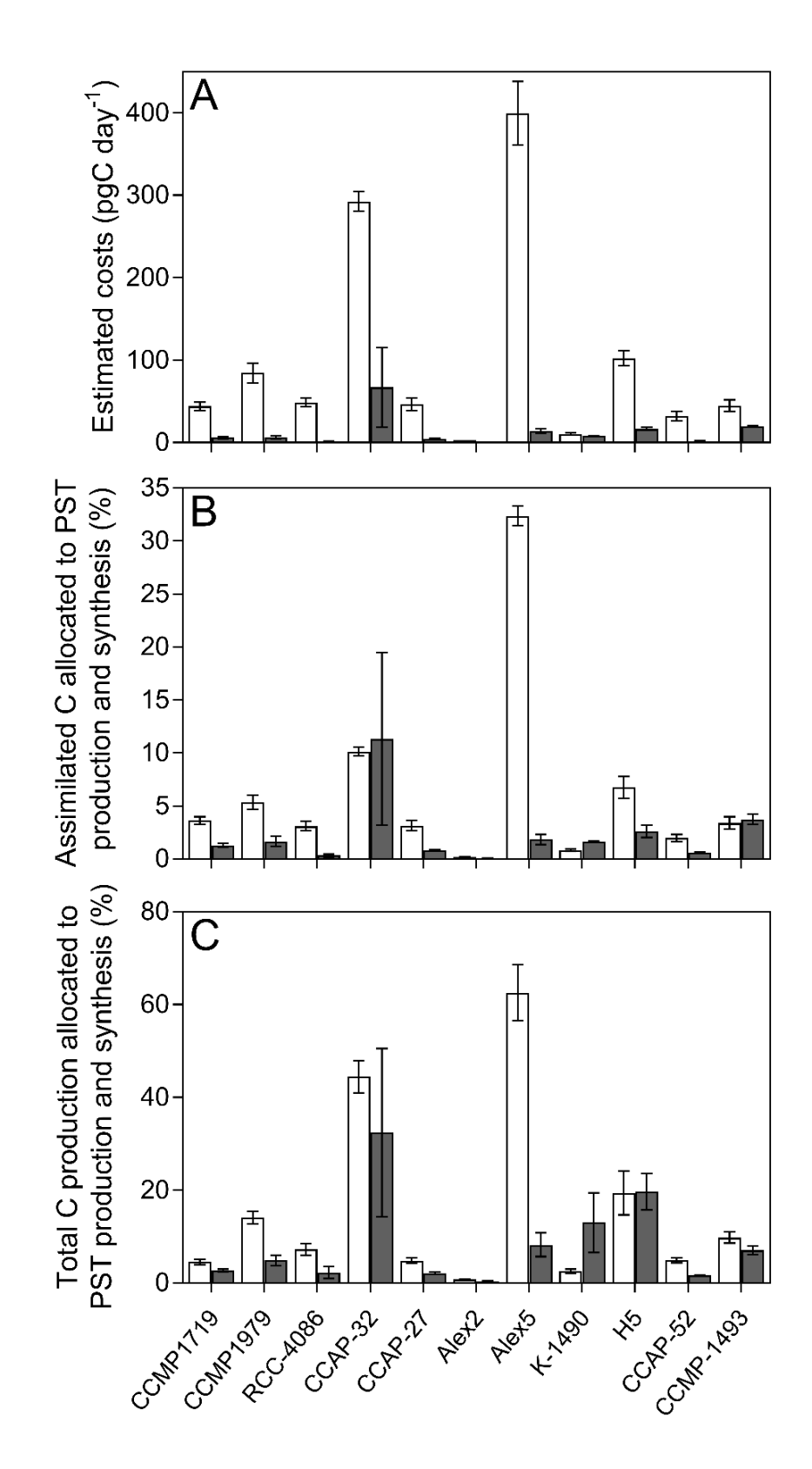


**Figure S3.** Costs of PST including estimated synthesis costs based on Chakraborty et al. (Chakraborty et al., 2018). **(A)** estimated synthesis costs of PST molecule and production in pgC cell^-1^ day^-1^. **(B)** estimated investment costs in terms of carbon in relation to total photosynthetic carbon uptake in each strain. **(C)** Estimated investment costs in terms of carbon to produce PST compared to total carbon production of the cell. HL is open bars, LL is closed bars, error bars represent standard error, N=3.


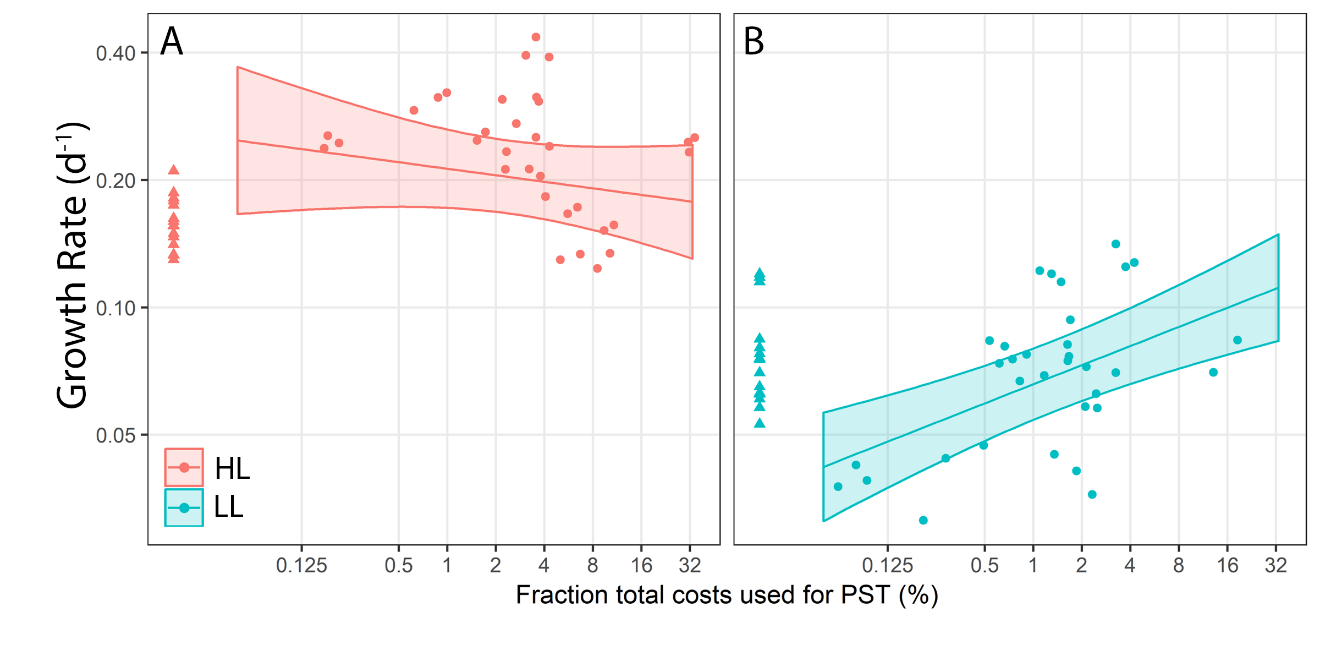


**Figure S4.** Estimated investment costs of PST production correlated with growth. This is including estimated synthesis costs calculated by Chakraborty et al. (2018). **(A)** is HL, **(B)** is LL. Circles are PST producers, triangles (placed close to the y-axis) are non-PST producers.


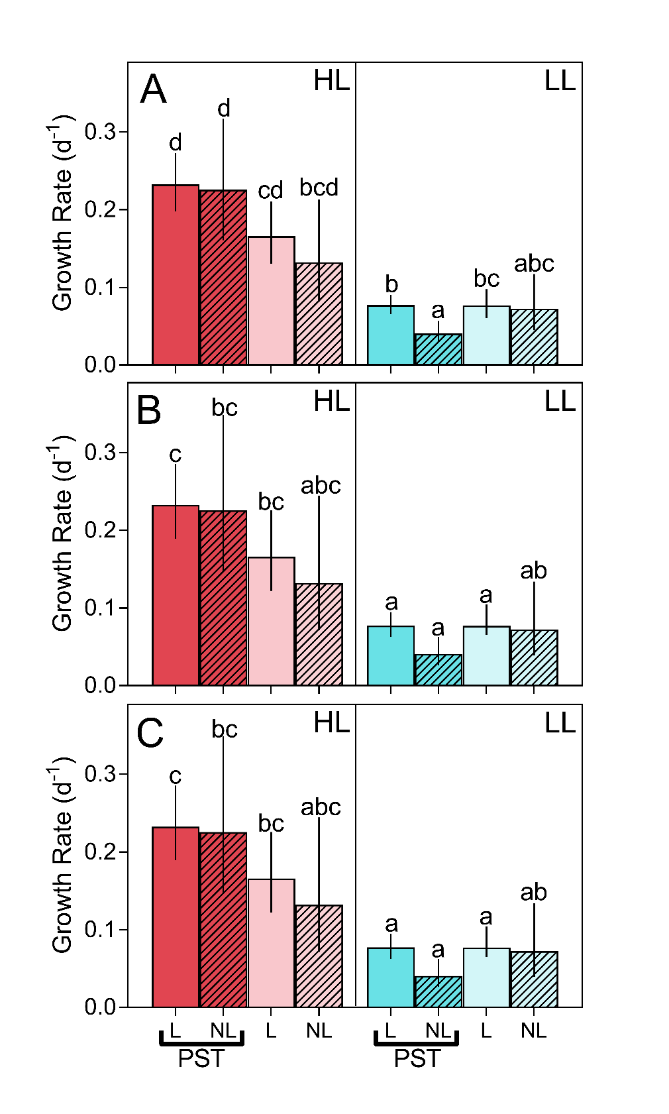


**Figure S5.** Estimated marginal means of the three ANCOVA models showing the 8 conditions. Shaded is non-lytic, no fill is lytic. **(A)** Model using PST content to express PST, **(B)** model using PST production to express PST, and **(C)** model using PST fraction to express PST. Error bars show the upper and lower limits, letters above error bars show significance differences between groups.

## Supplementary Tables

**Table S1. Estimated variance and parameter estimates for the final ANCOVA model of the effects of lytic toxicity and PST content (pg cell^-1^) on growth depending on light, presence/absence of PST, and lytic/non-lytic.**

| **Estimates for variance parameters of random effects** | | | | | | | |  |  | |  |
| --- | --- | --- | --- | --- | --- | --- | --- | --- | --- | --- | --- |
|  | Variance | Proportion  of variance | | |  |  | |  |  | |  |
| Strain | 0.022 | 40.3 | | |  |  | |  |  | |  |
| Light | 0.024 | 44.4 | | |  |  | |  |  | |  |
| Error | 0.008 | 15.3 | | |  |  | |  |  | |  |
| Total | 0.055 |  | | |  |  | |  |  | |  |
| **Parameter estimates, 95% confidence intervals and p-values** | | | | | | | | | | | |
| Parameter | | |  | Estimate | | | 2.5% | 97.5% | | p-value | |
| γ(HL) | | |  | -0.2464 | | | -0.3959 | -0.1298 | | 0.0039 | |
| γ(LL) | | |  | 0.1353 | | | -0.0188 | 0.2675 | | 0.1029 | |
| β(with PST) | | |  | -0.1685 | | | -0.2908 | -0.0914 | | 0.0019 | |
| β(without PST) | | |  | 0.0172 | | | -0.0904 | 0.1618 | | 0.8083 | |
| α(HL, with PST, non-lytic) | | |  | -0.5577 | | | -1.0562 | 0.0622 | | 0.1093 | |
| α(LL, with PST, non-lytic) | | |  | -3.6068 | | | -4.0639 | -3.0908 | | 0.0000 | |
| α(HL, without PST, non-lytic) | | |  | -2.0248 | | | -2.3745 | -1.6751 | | 0.0000 | |
| α(LL, without PST, non-lytic) | | |  | -2.6249 | | | -2.9746 | -2.2752 | | 0.0000 | |
| α(HL, with PST, lytic) | | |  | -1.5543 | | | -2.2674 | -0.9556 | | 0.0002 | |
| α(LL, with PST, lytic) | | |  | -3.7096 | | | -4.3825 | -3.1813 | | 0.0000 | |
| α(HL, without PST, lytic) | | |  | -1.7005 | | | -2.3227 | -0.8723 | | 0.0005 | |
| α(LL, without PST, lytic) | | |  | -2.4651 | | | -3.1123 | -1.6029 | | 0.0000 | |

**Table S2. Estimated variance and parameter estimates for the final statistical ANCOVA model of the effects lytic toxicity and PST production (pg cell^-1^ d^-1^) on growth, depending on light, PST presence/absence and lytic/non-lytic.**

| **Estimates for variance parameters of random effects** | | | | | | | |  |  | |  |
| --- | --- | --- | --- | --- | --- | --- | --- | --- | --- | --- | --- |
|  | Variance | Proportion  of variance | | |  |  | |  |  | |  |
| Strain | 0.056 | 63.9 | | |  |  | |  |  | |  |
| Light | 0.026 | 29.1 | | |  |  | |  |  | |  |
| Error | 0.006 | 7.1 | | |  |  | |  |  | |  |
| Total | 0.088 |  | | |  |  | |  |  | |  |
| **Parameter estimates, 95% confidence intervals and p-values** | | | | | | | | | | | |
| Parameter | | |  | Estimate | | | 2.5% | 97.5% | | p-value | |
| β | | |  | -0.1023 | | | -0.1800 | -0.0221 | | 0.0190 | |
| γ(HL) | | |  | -0.0259 | | | -0.2216 | 0.1300 | | 0.7868 | |
| γ(LL) | | |  | 0.3264 | | | 0.2012 | 0.4381 | | 0.0000 | |
| α(HL, with PST, non-lytic) | | |  | -1.4194 | | | -1.9544 | -0.7931 | | 0.0001 | |
| α(LL, with PST, non-lytic) | | |  | -3.2409 | | | -3.5945 | -2.8871 | | 0.0000 | |
| α(HL, without PST, non-lytic) | | |  | -2.0248 | | | -2.5247 | -1.5249 | | 0.0000 | |
| α(LL, without PST, non-lytic) | | |  | -2.6249 | | | -3.1248 | -2.1250 | | 0.0000 | |
| α(HL, with PST, lytic) | | |  | -1.9369 | | | -2.4433 | -1.3390 | | 0.0000 | |
| α(LL, with PST, lytic) | | |  | -3.2378 | | | -3.6278 | -2.8322 | | 0.0000 | |
| α(HL, without PST, lytic) | | |  | -2.3675 | | | -2.8657 | -1.8581 | | 0.0000 | |
| α(LL, without PST, lytic) | | |  | -3.1609 | | | -3.6754 | -2.6345 | | 0.0000 | |

**Table S3. Estimated variance and parameter estimates for the final ANCOVA model of the effects of lytic toxicity and PST, expressed as Fraction assimilated C allocated towards PST (%), on growth depending on light, PST presence/absence, and lytic/non-lytic**.

| **Estimates for variance parameters of random effects** | | | | | | | |  |  | |  |
| --- | --- | --- | --- | --- | --- | --- | --- | --- | --- | --- | --- |
|  | Variance | Proportion  of variance | | |  |  | |  |  | |  |
| Strain | 0.053 | 63.7 | | |  |  | |  |  | |  |
| Light | 0.025 | 29.2 | | |  |  | |  |  | |  |
| Error | 0.006 | 7.2 | | |  |  | |  |  | |  |
| Total | 0.084 |  | | |  |  | |  |  | |  |
| **Parameter estimates, 95% confidence intervals and p-values** | | | | | | | | | | | |
| Parameter | | |  | Estimate | | | 2.5% | 97.5% | | p-value | |
| β | | |  | -0.0974 | | | -0.1734 | -0.0168 | | 0.0231 | |
| γ(HL) | | |  | 0.0299 | | | -0.1292 | 0.1803 | | 0.7255 | |
| γ(LL) | | |  | 0.3180 | | | 0.2165 | 0.4246 | | 0.0000 | |
| α(HL, with PST, non-lytic) | | |  | -1.4520 | | | -1.8470 | -1.0612 | | 0.0000 | |
| α(LL, with PST, non-lytic) | | |  | -2.2688 | | | -2.7152 | -1.7952 | | 0.0000 | |
| α(HL, without PST, non-lytic) | | |  | -2.0248 | | | -2.5173 | -1.5323 | | 0.0000 | |
| α(LL, without PST, non-lytic) | | |  | -2.6249 | | | -3.1174 | -2.1323 | | 0.0000 | |
| α(HL, with PST, lytic) | | |  | -1.9070 | | | -2.4592 | -1.3440 | | 0.0000 | |
| α(LL, with PST, lytic) | | |  | -2.2806 | | | -2.7220 | -1.7994 | | 0.0000 | |
| α(HL, without PST, lytic) | | |  | -2.3401 | | | -2.8292 | -1.8311 | | 0.0000 | |
| α(LL, without PST, lytic) | | |  | -3.1323 | | | -3.6374 | -2.6062 | | 0.0000 | |

**References**

Chakraborty, S., Pančić, M., Andersen, K.H., and Kiørboe, T. (2018). The cost of toxin production in phytoplankton: the case of PST producing dinoflagellates. *The ISME journal***,** 1.

Daugbjerg, N., Hansen, G., Larsen, J., and Moestrup, Ø. (2000). Phylogeny of some of the major genera of dinoflagellates based on ultrastructure and partial LSU rDNA sequence data, including the erection of three new genera of unarmoured dinoflagellates. *Phycologia* 39(4)**,** 302-317.

Scholin, C.A., Herzog, M., Sogin, M., and Anderson, D.M. (1994). Identification of group‐and strain‐specific genetic markers for globally distributed *Alexandrium* (Dinophyceae). II. Sequence analysis of a fragment of the LSU rRNA gene. *Journal of phycology* 30(6)**,** 999-1011.
